# Haemoglobin levels may predict toxicities in patients on pelvic chemoradiation for carcinoma of the cervix—experience of a regional cancer centre

**DOI:** 10.3332/ecancer.2014.431

**Published:** 2014-05-19

**Authors:** Aparna Gangopadhyay, Joydeep Das, Partha Nath, Jaydip Biswas

**Affiliations:** 1 Department of Radiation Oncology Chittaranjan National Cancer Institute, 37 SP Mukherjee Road, Kolkata 700026, India; 2 Department of Medical Oncology, Chittaranjan National Cancer Institute, 37 SP Mukherjee Road, Kolkata 700026, India

## Abstract

**Background:**

Haemoglobin levels and tissue oxygenation influence tumour outcome in carcinoma cervix radiotherapy. The clinical impact of haemoglobin levels on acute normal tissue toxicity during radiation and interaction with chemotherapy in carcinoma of the cervix is underexplored. This paper aims to explore this issue.

**Methods:**

Treatment toxicity among 227 patients with squamous cell carcinoma of the cervix stages II B–IV A, receiving pelvic radiotherapy or chemoradiation at our institute, were studied prospectively. The baseline and weekly haemoglobin levels during treatment were recorded. Acute toxicities were recorded using Radiation Therapy Oncology Group (RTOG) acute toxicity and Common Terminology Criteria for Adverse Events (CTCAE) criteria, version 4. For the analysis, patients were divided into two groups, depending on nadir haemoglobin levels. A cut-off value for anaemia was selected at 12 gm/dL. Toxicity was compared between anaemic and non-anaemic groups.

**Results:**

Patients on chemoradiation and having haemoglobin levels >12 gm/dL suffered significantly higher dermatitis (two-tailed *p* value = 0.0288) and vaginal mucositis (two-tailed *p* value = 0.0187) of at least RTOG acute toxicity grade 2, compared with the anaemic group. In contrast patients receiving radiotherapy alone did not experience any significantly greater mucocutaneous toxicity if haemoglobin was >12 gm/dL. Anaemia had significantly greater impact on malaise and neutropenia (two-tailed *p* value <0.0001) of CTCAE grade 1 and above among chemoradiation patients, as opposed to those receiving radiotherapy alone (two-tailed *p* values = 0.0012 for neutropenia and 0.0422 for malaise).

**Conclusion:**

Haemoglobin values >12 gm/dL significantly worsen acute mucocutaneous toxicity in locally advanced cervical cancer patients receiving chemoradiation. Similar effects are not observed in the absence of chemotherapy.

## Introduction

Carcinoma of the cervix is a common cancer in women in developing countries, usually presenting at advanced stages where surgery is no longer a treatment option [1]. As a consequence, radiotherapy is the mainstay in the treatment of these patients.

From our perspective, a major health problem prevalent in the community is that of undernutrition, with anaemia being widely prevalent in the population. In India, the National Family Health Survey Data show that 55% of women of childbearing age are anaemic [2]. The World Health Organisation defines a value of 12 gm/dL as the cut-off value for anaemia in non-pregnant females above 15 years [3]. Although anaemia is defined variably in different oncology studies, 12 gm/dL was chosen as the cut-off point for anaemia in this paper to ensure that patients who were being deemed anaemic for the purposes of this paper were uniformly considered anaemic by National Community Health Programmes that follow WHO guidelines. An estimated third of carcinoma of the cervix patients at our institute have haemoglobin levels below 12 gm/dL at presentation, prior to start of treatment.

It is well known that anaemia is an adverse prognostic factor for disease outcomes in locally advanced carcinoma of the cervix, due to the association between anaemia and tissue hypoxia, which translates to impaired tumour radiation effect. However, the impact of anaemia on normal tissue acute toxicity and, therefore, treatment compliance among patients with carcinoma of the cervix remains an area of investigation.

The standard of care in locally advanced cervical carcinoma is pelvic chemoradiation. This treatment entails toxicity and may prove to be considerably morbid, especially when treatment volumes are large. Conformal radiotherapy techniques that help in reducing radiotherapy toxicity considerably cannot be offered to most patients in a limited-resources scenario.

The problems of undernutrition, anaemia, a large volume of disease, and intensive treatment, all in the setting of limited resources, continue to remain a widespread challenge for patients and health-care staff.

Under these circumstances, it was our endeavour to assess the impact of haemoglobin levels on acute treatment toxicity and treatment tolerance in these patients in our setting. The impact of haemoglobin levels on treatment toxicity was particularly relevant because it is a factor that may be easily corrected with appropriate interventions.

## Patients and methods

### Patients

A total of 227 patients with locally advanced squamous cell carcinoma of the cervix, ranging from stages II B to IV A (Table 1), who received either pelvic radiotherapy or pelvic chemoradiation in the Department of Radiation Oncology at our institute from October 2010 to November 2012, were assessed for incidence of acute treatment toxicity during pelvic chemoradiation and HDR brachytherapy. Haemoglobin levels were routinely monitored during the same period.

### Methods

The haemoglobin levels in all patients were recorded at the start of treatment and every week until completion of pelvic chemoradiation or radiotherapy. None of the patients had baseline haemoglobin levels below 10 gm/dL. Any patient needing transfusion for low haemoglobin levels after the start of treatment were excluded from the analysis. A total of 227 patients had been included in the study, of which 17 patients required blood transfusion during the course of treatment. These patients were excluded from analysis. The data of the remaining 210 patients were analysed (Table 2).

As per institutional protocol, all patients had haemoglobin values of 10 gm/dL or above during the entire treatment period.

Treatment toxicity was assessed weekly for the entire period of external beam radiotherapy and also at first brachytherapy appointment using Radiation Therapy Oncology Group (RTOG) acute toxicity criteria and Common Terminology Criteria for Adverse Events (CTCAE) version 4.02.

During data analysis, the nadir haemoglobin levels for each patient were taken into consideration, and incidence of acute treatment toxicity in relation to nadir haemoglobin levels was analysed.

### Treatment

#### Pelvic radiotherapy

Only those patients treated by conventional radiotherapy fields in telecobalt were studied to exclude any differences in toxicity that occur due to conformal planning or use of photons of variable energy.

#### External beam radiotherapy

All patients received 50 Gy in 25 fractions five days a week to the pelvic field by telecobalt. Pelvic radiotherapy was planned conventionally in all studied patients. As per institutional protocol, anterior-posterior and posterior-anterior portals were employed.

#### Brachytherapy

Intracavitary HDR brachytherapy (Ir^192^) sequentially followed external beam radiotherapy was received by all patients. A dose of 7 Gy x 4 fractions was employed.

#### Concomitant chemoradiation

During the period of pelvic radiotherapy, all patients who had adequate creatinine clearance received weekly concomitant Cisplatin chemotherapy at a dose of 40 mg/m^2^ weekly.

Any patient with contraindications to platinum therapy or those unwilling to receive chemotherapy received radiotherapy alone.

### Toxicity assessment

A weekly assessment of acute treatment-related toxicity based on RTOG acute toxicity criteria was made in all patients with respect to genitourinary and lower GI symptoms, dermatitis, and vaginal mucositis, although the latter is not easily amenable for assessment during treatment. In addition, neutropenia, vomiting, anorexia, malaise, urinary tract infection, and pelvic infection were assessed and graded weekly according to CTCAE version 4.02. For all patients, the highest grade of acute toxicity as per the RTOG scoring criteria or CTCAE grade suffered during treatment was taken into consideration for analysis.

### Statistical analysis

For analysis, patients were divided into two groups, depending on whether they were anaemic or non-anaemic. A cut-off value was selected at 12 gm/dL in accordance with the WHO definition of anaemia [4]. Any patient with nadir haemoglobin levels of 12 gm/dL or above was considered non-anaemic. All other patients were included in the anaemic group.

Fisher’s *t*-test (two sided) was used to assess any significant differences in acute treatment toxicity between anaemic and non-anaemic groups receiving either treatment modality (Table 3).

Statistical analysis was done using the Graph Pad QuickCalcs Web site: http://www.graphpad.com/quickcalcs/ contingency1 (accessed June 2013).

## Results

The ages of the patients receiving radiotherapy alone ranged from 39 to 68 years (median 54 years), and the group that received chemoradiation had a median age of 48 years (range 36–61 years).

Overall, the commonest stage at presentation was III B, with 78.41% of the patients having stage III B disease at presentation. Stage III B also comprised 88.70% of chemoradiation patients and 79.06% of those who received only radiotherapy. Moderately, differentiated squamous cell carcinoma was the commonest histological diagnosis comprising of 81.5% of the study population (Table 1).

Among 210 patients, 86 patients had received pelvic radiotherapy alone owing to comorbidities that prevented use of Cisplatin or refusing chemotherapy, and 124 patients received concurrent chemoradiation to the pelvis. Eighty-two patients had haemoglobin level of 12 gm/dL or higher. A total of 128 had haemoglobin levels between 10 and 12 gm/dL at baseline or at any time during therapy and comprised the anaemic group.

Grade 1 or 2 dermatitis or vaginal mucositis was more common among patients who received chemoradiation and had a haemoglobin level of >12 gm/dL (45% with haemoglobin >12 versus 29% and 66% versus 33%, respectively), but not in patients who only received radiation (35% versus 29% and 50% versus 59%, respectively). As expected, patients with lower haemoglobin reported more grade 1 or 2 malaise than patients with haemoglobin >12 gm/dL. Of patients treated with chemoradiation, 79% of those with a haemoglobin <12 reported grade 1 or 2 malaise versus 21% of those with a higher haemoglobin. Of patients treated with radiation only, 35% of those with a haemoglobin <12 reported grade 1 or 2 malaise versus 8% of those with higher a haemoglobin level.

Grade 1 or 2 neutropenia likewise was more common among patients with lower haemoglobin level. Sixty-three per cent of patients who received chemoradiation and had a nadir haemoglobin level of <12 gm/dL also developed grade 1 or 2 neutropenia during therapy versus 11% of those with a higher haemoglobin level. Forty-seven per cent of patients who only received only radiation and had a nadir haemoglobin level <12 developed grade 1 or 2 neutropenia versus 4% of those with higher haemoglobin.

Significance was tested for the association of these toxicities and haemoglobin levels and compared by treatment modality.

For patients receiving chemoradiation, the group having haemoglobin levels higher than 12 gm/dL suffered significantly higher incidence of dermatitis (two-tailed *p* value = 0.0288) and vaginal mucositis (two-tailed *p* value = 0.0187) of RTOG acute toxicity grade 2 or lower, compared with the anaemic group receiving same treatment. None of the patients in either group experienced grade 3 or 4 toxicity. There was very significant difference in the incidence of neutropenia and malaise between both groups. Patients who were anaemic experienced far more malaise (two-tailed *p* value <0.0001) and neutropenia (two-tailed *p* value <0.0001) of CTCAE grade 1 and above compared with patients who were not anaemic. No toxicity of grade 3 or 4 was noted.

The incidence of urinary tract infection was significantly higher in anaemic patients who received chemoradiation (two-tailed *p* value = 0.0207).

Treatment delays were higher in anaemic patients receiving chemoradiation, but statistical significance was not noted.

Among patients who received radiotherapy alone, there was no significant difference between acute radiation dermatitis and vaginal mucositis of grade 2 or lower experienced by patients who were anaemic as against those who had haemoglobin levels of 12 gm/dL or higher.

However, similar to the chemoradiation group, neutropenia was significantly higher in the anaemic patients receiving radiotherapy alone (two-tailed *p* value = 0.0012). Malaise was also significantly higher in anaemic patients who received radiotherapy alone (two-tailed *p* value = 0.0422), although the difference was not as pronounced as those who received chemoradiation.

Considering the anaemic patients from both treatment groups separately, there was no significant difference between treatment toxicity depending on whether anaemia was at baseline or at any time during therapy (Table 4).

On completion of treatment, a clinical response was found in 44/48 (91.7%) anaemic patients who received chemoradiation as opposed to 71/76 (93.4%) non-anaemic patients who had received the same treatment. This was found to be statistically non-significant.

Among patients who received radiotherapy alone, clinical response rates in anaemic patients and non-anaemic patients were 22/34 (64.7%) and 35/52 (67.3%), respectively, which was also not statistically significant.

The presence of anaemia according to the cut-off values chosen for this paper did not seem to affect disease response significantly.

Unfortunately, many patients studied had attended local hospitals and did not attend our institute for follow up in the long term, so it was not possible to assess for impact on late toxicity and recurrence patterns in this paper population.

It is relevant to state that a choice of a different and lower cut-off value for anaemia would possibly have yielded different results.

## Discussion

Iron deficiency anaemia is the most prevalent nutritional deficiency among Indian women. It is already established that anaemia is an adverse prognostic factor for treatment response among patients with carcinoma of the cervix receiving concurrent chemoradiation or radiotherapy alone [5]–[7]. Literature also points to worse outcomes among cervix cancer patients who require blood transfusions during treatment [8]. Unfortunately, anaemia and cervix cancer are both significant problems in women’s health care in our scenario, with quite a few patients requiring transfusions to maintain adequate haemoglobin levels during treatment.

While it is established that anaemia adversely impacts tumour outcomes during radiotherapy for carcinoma of the cervix, any impact of anaemia on normal tissue outcome is hardly available in literature for these patients.

Sufficient data in this regard from this part of the world, where both anaemia and cervix carcinoma are major health problems among women, are also not widely available.

There is evidence in the literature, mostly from other tumours, that anaemia influences acute radiation toxicity owing to the effect of hypoxia on acutely reacting normal tissue.

A number of clinical studies that reported radiation therapy outcomes in relation to anaemia have not shown consistent results, indicating the need for further exploration.

Henke *et al* [9] demonstrated lower acute radiation toxicity in anaemic subjects among a group of head and neck cancer patients receiving radiotherapy. The authors concluded that tissue hypoxemia could be the reason for less-severe acute reactions in anaemic patients. It is known that anaemia has adverse prognostic impact on tumour outcome in head and neck cancer radiotherapy similar to cervical cancer; whether the effects in relation to normal tissue toxicity for cervix cancer patients are similar is an area of interest.

Daly *et al* [10] studied acute radiation toxicity in locally advanced head and neck cancer patients treated by two different fractionation schedules. Normal tissue toxicity, as defined by the incidence of confluent mucositis was assessed and compared between two patient groups, one having anaemia and the other group without anaemia. The results showed no significant differences in normal tissue toxicity between both patient groups, although disease control was inferior among anaemic patients. Their study demonstrated absence of any difference in normal tissue toxicity due to anaemia.

In either of these studies on head and neck patients, chemotherapy was not added; the interaction between chemotherapy and anaemia could not be commented upon by the authors.

A study involving a large patient cohort from Spain, predominantly comprising breast cancer patients, explored the possible relationship between haemoglobin levels and incidence of acute radiation toxicity. This cohort comprised of patients who were treated by conventional radiotherapy and some patients had received concomitant chemotherapy, too. The correlation between absolute haemoglobin values and the degree of toxicity, and the possible relationship between haemoglobin concentration and the appearance of toxicity due to radiotherapy was not found to be significant. Differences in toxicity based on addition of chemotherapy or variable radiotherapy technique were not reported [11].

Radiosensitizers such as chemotherapy that improve therapeutic ratio by enhancing tumour control may potentially alter normal tissue toxicity. When these are combined with the added effect of hypoxia, it is possible that the interplay of these factor scould affect normal tissue toxicity in addition to tumour.

The discovery of the hypoxia inducible factor-1 alpha (HIF-1α) and its role in tumour behaviour and treatment response has increased interest in the signal molecules related to tumour hypoxia. Recent evidence suggests that radio sensitization does not just involve compounds that enhance the effect of radiation by mimicking oxygen, but explores compounds that target HIF-mediated signaling pathways eventually reducing radio resistance [12]. Considering that radiosensitizers have diverse mechanisms of action and that they may interact variably with hypoxia, a huge number of probabilities are thrown open in regard to tumour and normal tissue behaviour. In addition, differences in radiobiology of different tissues further widen probabilities.

The interesting finding in our patients was that patients with haemoglobin levels of 12 gm/dL or greater (*N* = 38) who received chemoradiation had more grade 1 or 2 dermatitis or vaginal mucositis than those with lower haemoglobin level (*N* = 24). This association was not seen in absence of Cisplatin. Evidently, hypoxia reduced normal tissue effects of Cisplatin.

In our study group most patients had conventional treatment fields. Conformal radiation techniques that improve therapeutic ratio of radiotherapy treatment play a prominent role in cervix cancer owing to proximity of target volume with organs at risk. However, the potential for greater normal tissue effects due to higher integral dose in the case of intensity-modulated radiotherapy also exists. The normal tissue effects in treatments with higher integral dose may differ from one individual to another on the basis of these interactions between tissue oxygenation and radiosensitizers.

## Conclusion

Radiation therapy aims at optimising therapeutic ratio by improving tumour cell kill and simultaneously reducing normal tissue toxicity. Widely exploited in the clinic is the knowledge of the radiobiology of different tissues, as well as use of agents that modify tumour response. The mechanisms of the interplay of factors that modify radiation effect are complex, and may lead to variable clinical outcomes. In the case of carcinoma of the cervix, there is established evidence that anaemia and hence hypoxia and radiosensitizers both have significant impact on tumour response. The normal tissue impact of the interaction between these factors has been relatively underexplored, although it is equally relevant in terms of clinical outcomes.

It was the aim of this study to explore the differences in normal tissue toxicity between groups of patients during treatment who had differences in two factors, chemotherapy and anaemia. The results from this study showed that the interaction of anaemia, and hence hypoxia with chemotherapy reduced acute radiation toxicity selectively in skin and vaginal mucosa, although an accurate estimate of vaginal mucositis during treatment is not easily made. Similar effects were not observed for other organs.

This paper results cannot be generalised owing to limitations in regard to patient number, treatment technique, the use of chemotherapy as the only radiosensitizer and also defining anaemia at a chosen cut-off value. Whether these results would vary for a larger cohort, for those receiving conformal radiotherapy and use of alternative radiosensitizers, warrants further investigation. Furthermore, this paper focused on acute toxicity only. Studies are needed to assess any difference in late effects and their clinical impact, if any.

## Conflicts of interest

The authors have no conflicts of interest to declare.

## Figures and Tables

**Table 1.** Composition of study patient population according to stage, age, and histology.

Stage	Total number of patients	Age ( in yrs)				Histology			
		30–40	41–50	51–60	61–70	Well diff squamous cell carcinoma	Mod diff squamous cell carcinoma	Poorly diff squamous cell carcinoma
II B	29	6	10	11	2	2	26	1
III A	8	3	3	2	0	0	5	3
III B	178	46	69	54	9	9	146	23
IV A	12	1	5	2	4	0	8	4

**Table 2.** Distribution of patients receiving either treatment modality according to disease stage.

Stage	Patients who received pelvic radiotherapy *n* = 86	Patients who received pelvic chemoradiation *n* = 124
II B	16	8
III A	0	2
III B	68	110
IV A	2	4

**Table 3.** Occurrence of acute toxicity among patients from either treatment group.

Toxicity during treatment	Pelvic radiotherapy (*n* = 86)				*p-*values	Pelvic chemoradiation (*n* = 124)				*p-*values
	Anaemic (*n* = 34)		Non-anaemic (*n* = 52)			Anaemic (*n* = 48)		Non-anaemic (*n* = 76)		
Dermatitis	10	29%	18	35%	NS	14	29%	34	45%	0.0288
Mucositis	20	59%	26	50%	NS	16	33%	50	66%	0.0187
Genitourinary	2	6%	0	0%	NS	4	8%	2	3%	NS
Lower gastrointestinal	4	12%	2	4%	NS	2	4%	0	0%	NS
Neutropenia	16	47%	2	4%	0.0012	30	63%	8	11%	<0.0001
Nausea	0	0%	0	0%	NS	34	70%	38	50%	NS
Vomiting	0	0%	0	0%	NS	4	8%	2	3%	NS
Anorexia	6	17%	4	8%	NS	30	62%	32	42%	NS
Malaise	12	35%	4	8%	0.0422	38	79%	16	21%	<0.0001
Urinary tract infection	0	0%	2	4%	NS	4	8%	0	0%	0.0207
Pelvic infection	2	6%	0	0%	NS	0	0%	0	0%	NS

NS = Not significant.

**Table 4.** Toxicity differences among anaemic patients, depending on baseline anaemia or at anytime during treatment.

Toxicity during treatment among anaemic patients (either at baseline or any time during therapy)	Pelvic radiotherapy			Pelvic chemoradiation		
	Total (*n* = 34)	Anaemia at baseline (*n* = 20)	Anaemia at any time during treatment (*n* = 14)	Total (*n* = 48)	Anaemia at baseline (*n* = 29)	Anaemia at any time during treatment (*n* = 19)
Dermatitis	10	6	4	14	5	9
Mucositis	20	8	12	16	9	7
Genitourinary	2	0	2	4	1	3
Lower gastrointestinal	4	2	2	2	0	2
Neutropenia	16	11	5	30	14	16
Nausea	0	0	0	34	11	23
Vomiting	0	0	0	4	3	1
Anorexia	6	2	4	30	9	21
Malaise	12	7	5	38	20	18
Urinary tract infection	0	0	0	4	2	2
Pelvic infection	2	1	1	0	0	0

## References

[1] Ferlay J, Soerjomataram I, Ervik M (2013). GLOBOCAN 2012 v1.0, Cancer Incidence and Mortality Worldwide: IARC Cancer Base 11 [Internet]. http://globocan.iarc.fr.

[2] International Institute for Population Sciences (IIPS) and Macro International (2007). National Family Health Survey (NFHS-3), 2005–06 India.

[3] WHO (2011). Haemoglobin concentrations for the diagnosis of anaemia and assessment of severity. Vitamin and Mineral Nutrition Information System. http://www.who.int/vmnis/indicators/haemoglobin.pdf.

[4] http://www.who.int/vmnis/indicators/haemoglobin.

[5] Obermair A (2003). Anemia before and during concurrent chemo radiotherapy in patients with cervical carcinoma: effect on progression-free survival. Int J Gynecol Cancer.

[6] Dunst J (2003). Anemia in cervical cancers: impact on survival, patterns of relapse, and association with hypoxia and angiogenesis. Int J Radiat Oncol Biol Phys.

[7] Yalman D (2003). Prognostic factors in definitive radiotherapy of uterine cervical cancer. Eur J Gynaecol Oncol.

[8] Santin AD (2003). Influence of allogeneic blood transfusion on clinical outcome during radiotherapy for cancer of the uterine cervix. Gynecol Obstet Invest.

[9] Henke M (2000). Blood hemoglobin level may affect radiosensitivity-preliminary results on acutely reacting normal tissues. Int J Radiat Oncol Biol Phys.

[10] Daly T (2003). The effect of anaemia on efficacy and normal tissue toxicity following radiotherapy for locally advanced squamous cell carcinoma of the head and neck. Radiother Oncol.

[11] Escó Barón R (2005). Hemoglobin levels and acute radiotherapy-induced toxicity. Tumori.

[12] Ghattass K (2013). Targeting hypoxia for sensitization of tumors to radio- and chemotherapy. Curr Cancer Drug Targets.

